# Prognostic value of SH3PXD2B (Tks4) in human hepatocellular carcinoma: a combined multi-omics and experimental study

**DOI:** 10.1186/s12920-021-00963-6

**Published:** 2021-04-28

**Authors:** Xiang Kui, Yan Wang, Cheng Zhang, Hai Li, Qingfeng Li, Yang Ke, Lin Wang

**Affiliations:** 1 Department of Pathology, The Second Affiliated Hospital of Kunming Medical University, Kunming, 650101 China; 2 Department of Hepatobiliary Surgery, The Second Affiliated Hospital of Kunming Medical University, Kunming, 650101 China; 3 Department of Hepatobiliary Surgery, The Sixth People’s Hospital of Chengdu, Chengdu, 610051 China; 4 School of Medicine, Kunming University, Kunming, 650214 China

**Keywords:** Clinicopathology, Hepatocellular carcinoma, Human Protein Atlas, Invasion, Omics, Proliferation, Recurrence, Survival, TCGA, SH3PXD2B

## Abstract

**Background:**

Hepatocellular carcinoma (HCC) is one of the most common and fatal cancers worldwide. HCC invasion and metastasis are crucial for its poor prognosis. SH3PXD2B is a scaffold protein and critical for intravascular and extravascular invasion and metastasis of various types of tumors. However, the role of SH3PXD2B in HCC progression remains unclear.

**Methods:**

The levels of SH3PXD2B mRNA transcripts in the TCGA database and SH3PXD2B protein expression in the Human Protein Atlas were analyzed. Furthermore, the levels of SH3PXD2B expression in clinical samples were analyzed by quantitative RT-PCR and immunohistochemistry. The potential association of the levels of SH3PXD2B expression with clinicopathological characteristics, overall survival (OS), and recurrence-free survival (RFS) of HCC patients was analyzed. The impact of SH3PXD2B silencing by shRNA-based lentivirus transduction on the proliferation and invasion of human HCC Hep3B and Huh7 cells was determined.

**Results:**

SH3PXD2B expression was up-regulated in HCC tissues in the TCGA and Human Protein Atlas as well as clinical samples, relative to that of non-tumor liver samples. The levels of SH3PXD2B expression in HCC tissues were significantly associated with higher HBV infection rate, higher HCC grades and TNM stages, higher Ki-67 expression, higher serum α-fetoprotein (AFP), a shorter OS and RFS of HCC patients. Functionally, SH3PXD2B silencing significantly inhibited the formation and function of invadopodia and the invasion of Hep3B and Huh7 cells, but did not affect their proliferation in vitro.

**Conclusions:**

Our data suggest that SH3PXD2B may promote the invasion and metastasis of HCC and be a valuable therapeutic target and biomarker for treatment and prognosis of HCC.

**Supplementary Information:**

The online version contains supplementary material available at 10.1186/s12920-021-00963-6.

## Background

Hepatocellular carcinoma (HCC) is a common and fatal liver cancer in the world and accounts for 841,000 new cases and 782,000 deaths a year [1]. Despite currently great advancements in both diagnosis and treatment of HCC [2–5], such as therapeutic procedures, new targeted drugs and PD-1/PD-L1 inhibitors, the therapeutic efficacy of HCC is not satisfied due to poor understanding of the molecular pathogenesis of HCC and the lack of valuable biomarkers for prognosis of HCC [6]. Hence, discovery of new therapeutic targets that are critical for the pathogenesis of HCC and reliable biomarkers for the prognosis of HCC will be of great significance.

It is well known that HCC invasion and metastasis are crucial for its poor prognosis. HCC invasion and metastasis are regulated by many factors, including the up-regulated epithelial-mesenchymal transition regulators, such as Twist1, Snai1, and Snai2 [7]. Furthermore, HCC invasion and metastasis are also positively regulated by the enhanced Rho-related signaling [8]. Hence, understanding the regulation of HCC invasion and metastasis may reveal new therapeutic targets and prognostic factors for HCC.

SH3 and PX domains 2B (SH3PXD2B) is also known as tyrosine kinase substrate with four SH3 domains (Tks4). It consists of the N-terminal phox homology domain, four Sr homology 3 domains, several proline-rich regions, and Src phosphorylation sites [9]. Our previous studies and those of others have shown that SH3PXD2B is an essential invadopodium scaffold protein and can stimulate actin polymerization, recruit membrane type-1 matrix metalloproteinase (MT1-MMP) to intracellular membranes, and increase Nox1-dependent reactive oxygen species (ROS) production [10, 11]. Actually, SH3PXD2B has been demonstrated to promote the intravascular and extravascular invasion and metastasis of human colon cancer, breast cancer, and melanoma cells [11–13]. However, there is no information on whether SH3PXD2B can regulate the invasion of HCC and can be a prognostic factor for HCC.

In the present study, we investigated the SH3PXD2B mRNA transcription and protein expression in The Cancer Genomics Atlas (TCGA) and the Human Protein Atlas, a proteome database, as well as clinical HCC specimens, and analyzed their association with overall survival (OS) and recurrence-free survival (RFS) of HCC patients. Furthermore, we explored the impact of SH3PXD2B silencing on the proliferation and invasion of HCC cells in vitro. Our data indicated that SH3PXD2B was a valuable biomarker for the prognosis of HCC.

## Materials and methods

### Bioinformatics analysis

The levels of SH3PXD2B mRNA transcripts in HCC and non-tumor liver tissues in the TCGA database were analyzed by the GEPIA2 (http://gepia2.cancer-pku.cn/) [15] and the potential association of SH3PXD2B expression with the race, age, grade, stage, and other clinicopathological characteristics of HCC patients as well as their OS and RFS were analyzed after stratification, based on the median value of SH3PXD2B expression levels. In addition, the levels of SH3PXD2B expression in immunohistochemically analyzed 6 HCC and 3 non-tumor liver tissue sections available in the Human Protein Atlas database (https://www.proteinatlas.org/) [14] were analyzed using the Image-Pro Plus 6.0, Media, Cybernetics and expressed as the mean optical density (MOD) [15]. The detailed information on the subjects and analysis is shown in the Additional file 1. The OS was defined as the period from the surgery day to the death day or the last contact day. The RFS was defined as the period from the surgery day to the tumor recurrence day.

### HCC tissue samples

A total of 89 HCC patients were recruited at the Second Affiliated Hospital of Kunming Medical University from January 2016 to December 2018. Their HCC and adjacent non-tumor liver tissue samples were collected when they underwent a hepatectomy to remove the tumors. Those patients were diagnosed, based on clinical symptoms, laboratory and radiological examinations as well as pathological confirmation. HCC patients with transcatheter arterial chemoembolization, chemotherapy, or radiotherapy before surgery were excluded. All patients were regularly followed-up until December 2019. All patients have signed the written informed consent and the study was approved by the Institutional Review Boards of the Second Affiliated Hospital of Kunming Medical University. Twenty-eight pairs of fresh tissue specimens were snap-frozen in liquid nitrogen and used for qRT-PCR in our study. Other specimens were fixed in formalin and paraffin-embedded for immunohistochemistry.

### RNA extraction and quantitative real-time PCR analysis

Total RNAs were extracted from fresh tissue samples using the Trizol reagent (Invitrogen, Carlsbad, CA, USA) and reversely transcribed into cDNA using the PrimeScript RT reagent Kit (TaKaRa, Japan) after treatment with RNase-free DNase I (Promega, USA). The relative levels of SH3PXD2B mRNA transcripts in individual samples were quantified in triplicate by qRT-PCR using the SYBR Premix Ex Taq (Takara) and specific primers F: AGATTCTCTTCAGACGAA and R: GCCTTACAGTATTCATCAA for SH3PXD2B; and F: TGTTGCCATCAATGACCCCT and R: TCGCCCCACTTGATTTTGGA for GAPDH. The data were analyzed by the 2-^ΔΔ^CT method.

### Immunohistochemistry (IHC)

The levels of SH3PXD2B protein expression in HCC and adjacent tissues were determined by IHC. Briefly, the HCC and adjacent tissue sections (4 μm) were dewaxed, rehydrated, and immersed in 3% methanol to inactivate endogenous peroxidase. After being washed, the sections were blocked with 5% BSA in TBST and incubated with rabbit polyclonal antibodies against SH3PDX2B (PA5-57673, Invitrogen, 1:500 dilution) at 4 °C overnight. The bound antibodies were detected with horseradish peroxidase (HRP)-conjugated goat anti-rabbit IgG (Maixin Bio, Fujian, China) and visualized by diaminobenzidine (DAB). The intensity of anti-SH3PXD2B protein staining was determined as a MOD by Image-Pro Plus 6.0 (Media, Cybernetics). A non-stained region was selected and served as the background.

### Cell culture

Human HCC Hep3B and Huh7 cells were obtained from the Kunming Cell Bank, Kunming Institute of Zoology, Chinese Academy of Sciences (Kunming, China) and identified by short tandem repeat (STR) analysis. Hep3B and Huh7 cells were cultured in Dulbecco’s modified Eagle’s Medium (DMEM) supplemented with 10% fetal bovine serum (FBS), 100 units/ml of penicillin and 100 μg/ml of streptomycin at 37 °C in a humidified incubator with 5% CO_2_.

### Generation of lentiviruses and transduction

The SH3PXD2B-specific small hairpin RNA (sense: 5′-GGTGCCCAACAAGCATTATGT-3′) and control (sense: 5′-TTCTCCGAACGTGTCACGT-3′) shRNAs were synthesized and cloned into the plasmid of pGLVU6/GFP. The generated plasmids, together with packaging plasmids, were co-transfected into 293T cells using lipofectamine 3000 (Invitrogen) to generate different types of recombinant lentivirus virions. HCC cells were transduced with each type of lentivirus at a multiplicity of infection (MOI) of 5 and cultured in the presence of 4 μg/ml of puromycin for 7 days to generate stably SH3PXD2B-silenced or control cells. The efficacy of SH3PXD2B silencing was determined by Western blot.

### Western blot assay

The relative levels of SH3PXD2B to β-tubulin proteins were determined by Western blot. Briefly, different groups of cell lysates (30 μg/lane) were separated by SDS-PAGE on 12% gels and transferred onto PVDF membranes. After being blocked, the membranes were probed with rabbit polyclonal anti-SH3PDX2B antibodies (PA5-57673, Invitrogen), anti-β-tubulin antibodies (PA5-16863, Invitrogen). The bound antibodies were detected with HRP-conjugated goat anti-rabbit IgG (H + L) (G-21234, Invitrogen) and visualized using the enhanced chemiluminescent reagents. The data were analyzed by densitometry using the Image J software.

### Cell proliferation curves

The different groups of HCC cells (1 × 10^5^ cells/well) were cultured in triplicate for varying periods and the numbers of viable cells per well were determined after trypan blue staining.

### Transwell invasion assays

The different groups of HCC cells (1 × 10^5^ cells/well) were cultured in the serum-free medium in the upper chambers that had been coated with ECMatrixTM. The lower chambers were filled with 10% FBS DMEM medium. After cultured for 24 h, the cells on the top surface of the upper chambers were removed, and the cells on the bottom surface were stained with crystal violet. The numbers of invaded cells per field were counted and 10 random fields at 200 × magnification were analyzed.

### Invadopodium formation assays

The impact of SH3PXD2B silencing on the invadopodium formation was determined by invadopodium formation assay, as described previously [16]. Briefly, the different groups of HCC cells were cultured on coverslips coated with gelatin (50 μg/ml, G1890, Sigma-Aldrich, St. Louis, MO). Eight hours later, the cells were fixed for immunofluorescence using rabbit anti-Cortactin [EP1922Y] (1:1,000, ab81208, Abcam, Cambridge, MA) and Alexa Fluor® 488-labeled goat anti-rabbit IgG (2 μg/ml, ab150081, Abcam) as well as Alexa Fluor™ 568-labeled Phalloidin (1:200, A12380, Invitrogen). Subsequently, the cells were nuclear-stained with DAPI (Invitrogen). The fluorescent signals were captured under a confocal microscope (Nikon A1, Tokyo, Japan) and analyzed using NIS-Elements Free Viewer Ver4.20.00.

### In situ zymography

The effects of SH3PXD2B silencing in HCC cells on the invadopodium-associated matrix degradation were analyzed by in situ zymography [16]. Briefly, the different groups of cells were cultured on coverslips coated with FITC-conjugated gelatin (M1303-1, BioVision, Milpitas, CA). Eight hours later, the cells were fixed and stained with Alexa Fluor™ 568-labeled Phalloidin (1:200, A12380, Invitrogen) and nuclear-stained with DAPI. The fluorescent signals were captured under a confocal microscope (Nikon A1, Tokyo, Japan) and analyzed using NIS-Elements Free Viewer Ver4.20.00.

### Statistical analysis

Data analyses were performed using IBM SPSS Statistics 22.0 (IBM, Armonk, NY, USA). Data are expressed as mean ± standard deviation or case number. The difference between groups was analyzed by the Student’s t-test, paired t-test, Cochran-Armitage trend test, and chi-square test where applicable. HCC patients were stratified into the higher and lower SH3PXD2B mRNA transcription groups, based on the median value. Their OS and RFS were estimated by the Kaplan–Meier method and analyzed by the log-rank test. Similarly, HCC patients were stratified into the higher and lower groups, based on the least *P* value for their OS determined using the X-Tile software (Yale University, New Haven, CT). Statistical significance was declared when a *P* value < 0.05.

## Results

### Up-regulated SH3PXD2B expression exists in HCC tissues

**Fig. 1:**
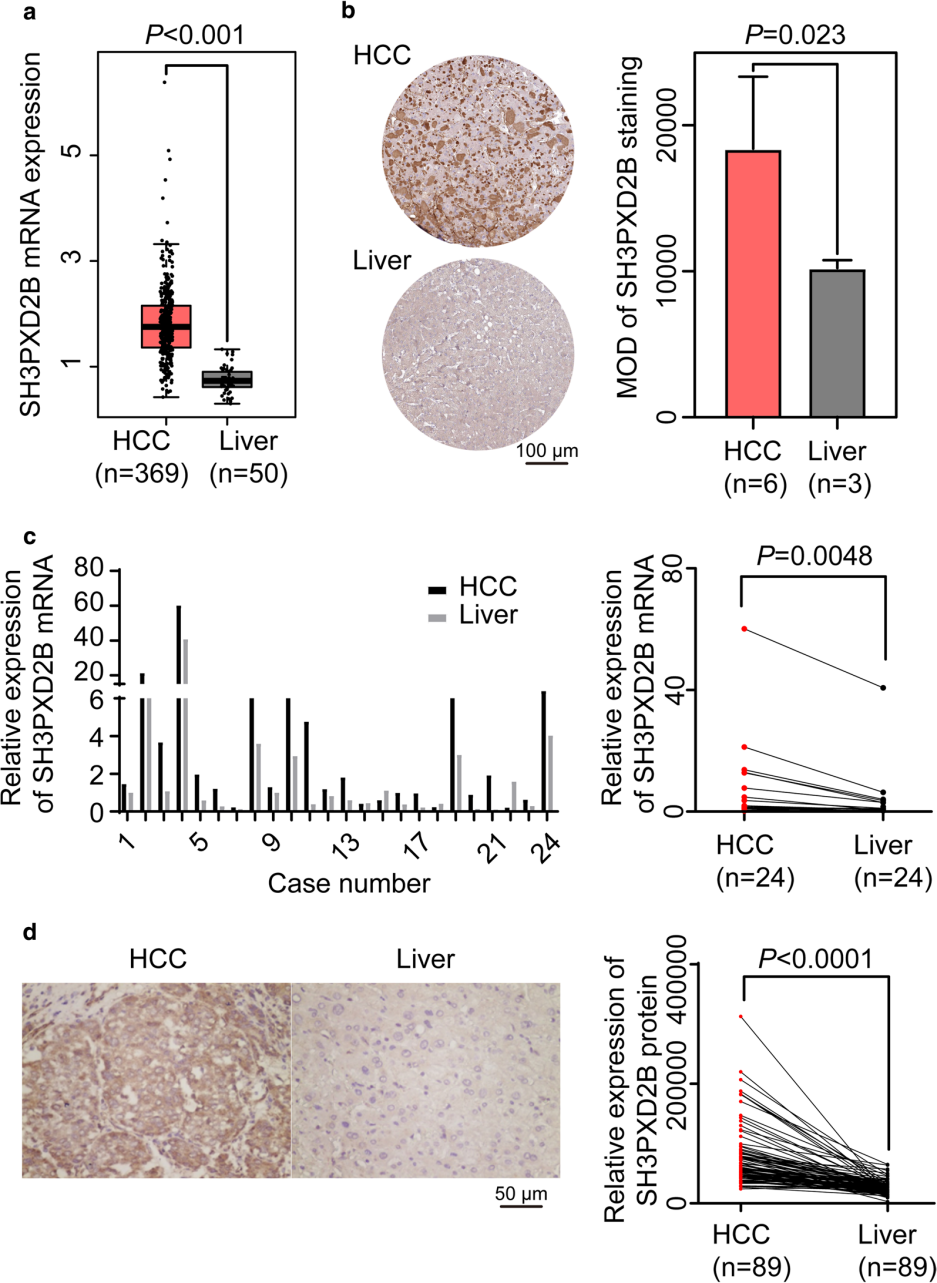
SH3PXD2B expression is up-regulated in HCC tissues. The levels of SH3PXD2B mRNA transcripts and protein expression in HCC tissues of the TCGA database (**a**) and the Human Protein Atlas (**b**) were analyzed. **c** The relative levels of SH3PXD2B mRNA transcripts in 24 pairs of freshly surgical HCC and paired non-tumor liver tissues were analyzed by qRT-PCR. **d** Immunohistochemical analysis of 89 pairs of HCC and non-tumor liver samples. Data are representative images (magnification × 200) or expressed as the mean values in individual samples or mean ± SD of each group

To investigate the clinical relevance of SH3PXD2B expression in human HCC, we analyzed the SH3PXD2B expression in the TCGA and Human Protein Atlas. The levels of *SH3PXD2B* mRNA transcripts in HCC tissues of the TCGA database were significantly higher than that in non-tumor liver tissues (*P* < 0.001, Fig. 1a). Similarly, the levels of SH3PXD2B protein expression in HCC tissues of the Human Protein Atlas were also significantly higher than that in non-tumor liver tissues (*P* = 0.023, Fig. 1b). Furthermore, qRT-PCR analysis of 24 pairs of fresh clinical HCC samples indicated that the relative levels of SH3PXD2B mRNA transcripts significantly increased in HCC tissues, compared with that in non-tumor liver tissues (*P* = 0.0048, Fig. 1c). Moreover, IHC characterization revealed that the SH3PXD2B protein was expressed in the cytoplasm of hepatocytes and the levels of SH3PXD2B expression in 89 HCC specimens were significantly higher than that in the paired non-tumor liver samples (*P* < 0.0001, Fig. 1d and Additional file 1: Fig. S1). Hence, up-regulated SH3PXD2B expression occurred in HCC tissues and may contribute to the progression of HCC.

### Up-regulated SH3PXD2B expression is associated with poor prognosis of HCC patients

**Fig. 2:**
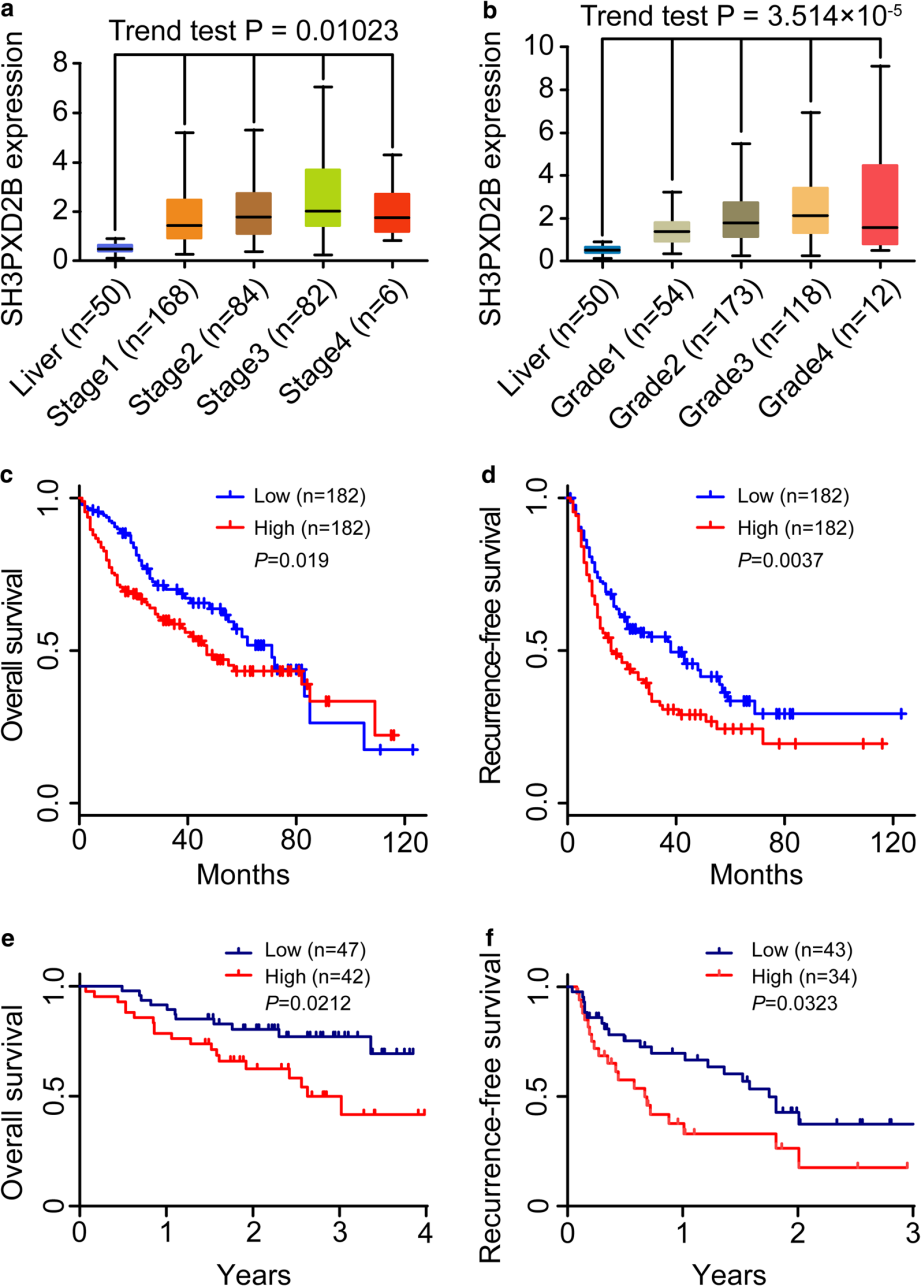
Higher levels of SH3PXD2B expression are associated with shorter survival periods of HCC patients. **a**, **b** All HCC patients were stratified, according to their TNM stages or pathological grades and the levels of SH3PXD2B mRNA transcripts were analyzed. Data are shown as the box and whisker plot, and the lines indicate the means of each group. **c**, **d** HCC patients were stratified, according to the median value of SH3PXD2B mRNA transcripts and their overall survival (OS) and recurrence-free survival (RFS) were analyzed by the Kaplan–Meier method and the log-rank test. **e**, **f** The OS and RFS of 89 HCC patients were analyzed after stratification of them into higher and lower SH3PXD2B expression with the least *P* value of OS, determined using the X-Tile software

To explore the relationship between SH3PXD2B expression and HCC clinicopathologic parameters, we analyzed the liver hepatocellular carcinoma (LIHC) data in the TCGA database and found the levels of SH3PXD2B mRNA transcripts were positively associated with the pathological grades and TNM stages of HCC samples (both *P* < 0.05, Fig. 2a, b). Based on our recruited 89 HCC patients, higher levels of SH3PXD2B protein expression were significantly associated with positive HBsAg detection (*P* = 0.004), higher Edmondson-Steiner grades (*P* = 0.023), higher TNM stages (*P* = 0.037), higher Ki-67 expression (*P* = 0.040), and higher AFP expression (*P* = 0.030), but not with other characteristics in this population (Table 1). More importantly, higher levels of SH3PXD2B expression were associated significantly with shorter OS and RFS periods than those with lower SH3PXD2B expression in HCC patients in the TCGA database and patients we collected in our hospital (all *P* < 0.05, Fig. 2c–f). Our results indicated that up-regulated SH3PXD2B expression was associated with a poor prognosis of HCC patients.

**Table 1:** Association between the clinicopathologic characteristics and the SH3PXD2B expression levels in HCC patients (n = 89)

Characteristics	n	SH3PXD2B expression		χ^2^	*P* value
		High	Low		
*Gender*					
Male	77	35	42	0.691	0.406
Female	12	7	5		
*Age (years)*					
≥ 60	23	8	15	1.916	0.166
< 60	66	34	32		
*HBsAg*					
Positive	71	39	32	8.436	0.004
Negative	18	3	15		
*Liver fibrosis*					
0	2	2	0	2.535	0.638
1	19	8	11		
2	14	7	7		
3	7	3	4		
4	47	22	25		
*Liver cirrhosis*					
Yes	47	22	25	0.006	0.939
No	42	20	22		
*Tumor size (cm)*					
≥ 5	56	27	29	0.063	0.801
< 5	33	15	18		
*Tumor number*					
Single	66	31	35	0.005	0.944
Multiple	23	11	12		
*Microvascular invasion*					
M0	36	17	19	0.860	0.651
M1	37	19	18		
M2	16	6	10		
*Macrovasucular invasion*					
Yes	16	9	7	0.642	0.432
No	73	33	40		
*Edmondson–Steiner Grade*					
I–II	25	7	18	5.138	0.023
III–IV	64	35	29		
*TNM stage*					
I–II	70	29	41	4.369	0.037
III–IV	19	13	6		
*Ki-67*					
≥ 30%	47	27	20	4.203	0.040
< 30%	42	15	27		
*AFP*					
≥ 400 ng/ml	36	22	14	4.700	0.030
< 400 ng/ml	53	20	33		
*CA125*					
≥ 35U/ml	7	1	6	3.301	0.114
< 35U/ml	82	41	41		
*CA199*					
> 35U/ml	8	2	6	1.737	0.273
≤ 35U/ml	81	40	41		
*TP53 mutation*					
Yes	52	23	29	0.440	0.507
No	37	19	18		

### SH3PXD2B silencing inhibits the invasion of HCC cells

**Fig. 3:**
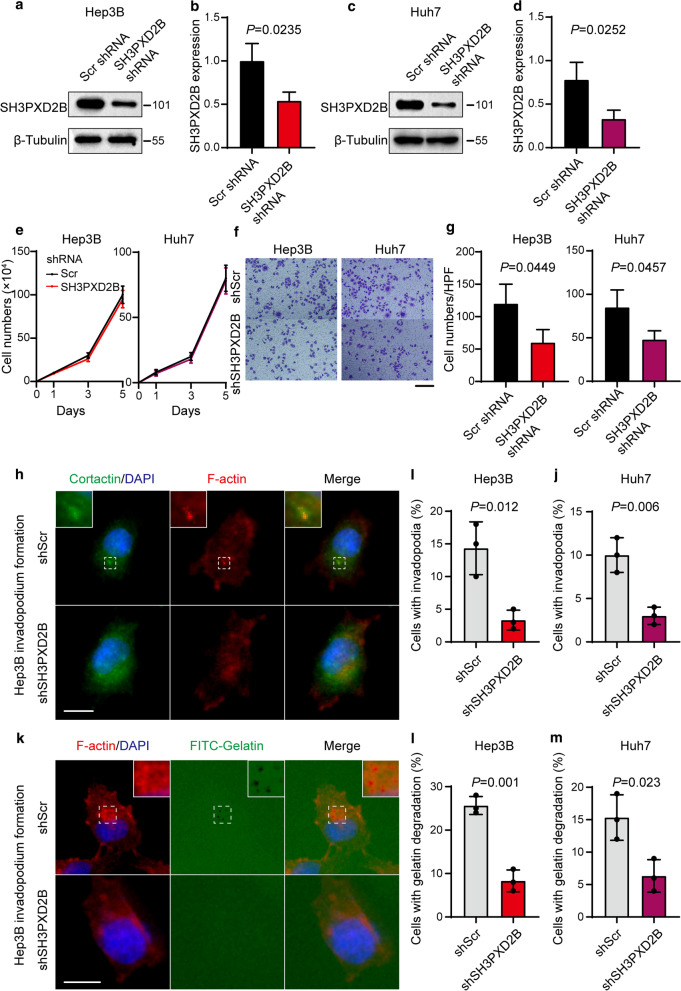
SH3PXD2B silencing inhibits the invasion, but not proliferation of Hep3B and Huh7 cells. Hep3B and Huh7 cells were transduced with lentivirus for expression of SH3PXD2B-specific shRNA or control shRNA (Scr). The relative levels of SH3PXD2B to the control β-tubulin protein expression in different groups of Hep3B (**a**, **b**) and Huh7 (**c**, **d**) cells were determined by Western blot. **e** The dynamic proliferation of Hep3B and Huh7 cells was determined at the indicated time points. **f**, **g** The invasion of different groups of Hep3B and Huh7 cells was examined by transwell invasion assays. **h**–**j** The formation of invadopodia in different groups of Hep3B and Huh7 cells was examined by immunofluorescence assays. **k**–**m** The function of invadopodia in different groups of Hep3B and Huh7 cells was examined by in situ zymography. A total of 150 cells per group were analyzed by two researchers in a blinded manner. Data are representative images or expressed as the mean ± SD of each group from three separate experiments. Bar scale in F = 100 μm. Bar scales in H and K = 20 μm

Because HCC invasion and metastasis are crucial for poor outcomes of HCC patients, we further investigated the function of SH3PXD2B in regulating the progression of HCC by testing the impact of SH3PXD2B silencing on the proliferation and invasion of HCC cells in vitro. We found that transduction of HCC Hep3B and Huh7 cells with lentivirus for expression SH3PXD2B-specific shRNA significantly decreased the relative levels of SH3PXD2B expression by 52%-64%, compared with the control cells (both *P* < 0.05, Fig. 3a–d). Longitudinal analysis indicated that SH3PXD2B silencing did not alter the dynamic proliferation of both Hep3B and Huh7 cells in vitro (both *P* > 0.05, Fig. 3e). In contrast, transwell invasion assays revealed that SH3PXD2B silencing significantly reduced the number of invaded HCC cells, compared with that of the control cells (both *P* < 0.05, Fig. 3f, g). Furthermore, SH3PXD2B silencing significantly inhibited the formation and in situ gelatin degradation function of invadopodia in HCC cells (all *P* < 0.05, Fig. 3h–m). Therefore, SH3PXD2B promoted the invasion of HCC cells, but not their proliferation in vitro.

## Discussion

In this study, we found that SH3PXD2B expression was significantly up-regulated in HCC tissues, relative to that of non-tumor liver tissues and the up-regulated SH3PXD2B expression was significantly associated with advanced stages, higher grades of HCC as well as shorter OS and RFS periods of HCC patients. Our results extended previous observations of up-regulated SH3PXD2B expression in human melanoma [13] and that up-regulated Tks5, another Tks adaptor protein similar to SH3PXD2B [9], expression has prognostic potential for human melanoma, breast cancer, glioma, lung cancer, and prostate cancer [13, 17–20]. To the best of our knowledge, these were not reported in the literature. Our novel findings suggest that SH3PXD2B may act as an oncogenic factor to promote the progression of HCC, such as invasion and metastasis, contributing to poor outcomes of HCC patients. Hence, SH3PXD2B may be a therapeutic target for development of therapies for HCC and reliable biomarker for prognosis of HCC patients.

To understand the function of SH3PXD2B in the progression of HCC, we tested the impact of SH3PXD2B silencing on the dynamic proliferation and invasion of human HCC Hep3B and Huh7 cells in vitro. After successfully silencing SH3PXD2B expression, we found that SH3PXD2B silencing did not affect the dynamic proliferation of both HCC cells in vitro. However, SH3PXD2B silencing significantly inhibited the invasion of both HCC cells in vitro. Indeed, SH3PXD2B is critical for functional invadopodium formation, which is a key step for cancer cell invasion [10, 16]. Furthermore, SH3PXD2B phosphorylation by Src can interact with NoxA1 to enhance the Nox1-dependent ROS production and invadopodium formation [11]. Functionally, SH3PXD2B can recruit membrane type-1 matrix metalloproteinase (MT1-MMP) to the incomplete podosomes to activate MMP2 and MMP9, leading to matrix degradation and cancer cell invasion [10]. Given that HCC invasion and metastasis are important factors for poor prognosis, genetic and functional inhibition of SH3PXD2B may be valuable for minimizing HCC metastasis.

Interestingly, we found that SH3PXD2B silencing did not significantly alter the dynamic proliferation of HCC cells in two-dimensional cultures. Such data were consistent with a previous study in melanoma [13]. However, the same study indicated that SH3PXD2B silencing inhibited the proliferation of melanoma cells in the collagen I-based three-dimensional cultures, as well as both primary and metastatic tumor growths in vivo [13]. Similar data were observed in Tks5 silencing [21, 22]. Accordingly, such findings suggest that SH3PXD2B or Tks5 may modulate the tumor environment, perhaps by creating physical space for tumor cell growth or possessing growth factors by proteases, to promote tumor growth. Furthermore, it is important to note that we did not completely disrupt the *SH3PXD2B* expression in HCC cells and therefore it is possible that a limited amount of SH3PXD2B expression may be sufficient for maintaining HCC cell growth. We are interested in further investigating whether SH3PXD2B knockout by CRISPR/Cas9 can modulate the growth and metastasis of HCC in vitro and in vivo in the future studies.

Chronic HBV infection, particularly for the related liver fibrosis and cirrhosis, is a risk factor for the development and prognosis of HCC [23–25] and laminin is a key factor for liver fibrosis and cirrhosis [26]. In this study, we found that up-regulated SH3PXD2B expression was associated with positive HBsAg detection in this population. Interesting, a recent study has shown that laminin augments the expression of SH3PXD2B, Tks5, cortactin, and MT1-MMP [27]. It is possible that chronic HBV infection-related fibrosis and cirrhosis may up-regulate the expression of laminin that enhances SH3PXD2B expression, promoting HCC cell invasion and metastasis. We will test this hypothesis in the future studies.

In conclusion, our data indicated the up-regulated SH3PXD2B expression in HCC was significantly associated with progression of HCC and a shorter survival of HCC patients. Furthermore, we found that SH3PXD2B silencing inhibited the invasion of human HCC cells in vitro. Our novel findings suggest that SH3PXD2B may be a therapeutic target for inhibition of HCC metastasis and higher levels of SH3PXD2B expression may be a biomarker for prognosis of HCC patients. We recognized that our study had limitations, including the relatively small sample size, without studies on the precise functions of SH3PXD2B in the progression of HCC and a retrospective analysis. Therefore, further prospective validation of the prognostic value of SH3PXD2B expression in the survival of HCC patients are warranted.

## Supplementary Information

**Additional file 1.** **Supplementary Methods. Supplementary Table 1.** The demographic information and mean optical density (MOD) of anti-SH3PXD2B staining in individual sections from the Human Protein Atlas database. **Supplementary Figure 1.** Up-regulated SH3PXD2B protein expression in human HCC tissues. **Supplementary Figure 2.** The original immunoblots using anti-SH3PXD2B in Figure 3.

## Data Availability

The demographic, clinical, and transcriptomic data are available in TCGA database on http://cancergenome.nih.gov/. The proteomic data are available in The Human Protein Atlas on https://www.proteinatlas.org/. The demographic, clinical, and genetic data from our clinical samples that support the omics findings of this study are available in Table 1.

## Abbreviations

HCC: Hepatocellular carcinoma
TCGA: The Cancer Genomics Atlas
Tks4: Tyrosine kinase substrate with four SH3 domains
SH3PXD2B: SH3 and PX domains 2B
MT1-MMP: Membrane type-1 matrix metalloproteinase
ROS: Reactive oxygen species
OS: Overall survival
RFS: Recurrence-free survival
GAPDH: Glyceraldehyde-3-phosphate dehydrogenase
HRP: Horseradish peroxidase
DAB: Diaminobenzidine
