# Supplementary material for: Prognostic value of SH3PXD2B (Tks4) in human hepatocellular carcinoma: a combined multi-omics and experimental study
Source: BMC Med Genomics. 2021 Apr 28;14:115. doi: 10.1186/s12920-021-00963-6 (PMC8080318; doi:10.1186/s12920-021-00963-6)
Supplement: Supplementary file 1 — Additional file 1. Supplementary Methods. Supplementary Table 1. The demographic information and mean optical density (MOD) of anti-SH3PXD2B staining in individual sections from the Human Protein Atlas database. Supplementary Figure 1. Up-regulated SH3PXD2B protein expression in human HCC tissues. Supplementary Figure 2. The original immunoblots using anti-SH3PXD2B in Figure 3. [file 12920_2021_963_MOESM1_ESM.docx]

Prognostic value of SH3PXD2B (Tks4) in human hepatocellular carcinoma: a combined multi-omics and experimental study

Xiang Kui^1*^, Yan Wang^1*^, Cheng Zhang^2,3*^, Hai Li^4*^, Qingfeng Li^1*^, Yang Ke^2ξ^, Lin Wang^2ξ^

*These authors contributed equally to this work.

ξCorrespondence

Dr. Yang Ke, Department of Hepatobiliary Surgery, the Second Affiliated Hospital of Kunming Medical University, Kunming 650101, China. Tel: +86 15808875159

E-mail: keyang1218@126.com

and

Dr. Lin Wang, Department of Hepatobiliary Surgery, the Second Affiliated Hospital of Kunming Medical University, Kunming 650101, China. Tel: +86 13888294845

E-mail: linwang0705@126.com

1. Department of Pathology, the Second Affiliated Hospital of Kunming Medical University, Kunming 650101, China.

2. Department of Hepatobiliary Surgery, the Second Affiliated Hospital of Kunming Medical University, Kunming 650101, China.

3. Department of Hepatobiliary Surgery, the Sixth People’s Hospital of Chengdu, Chengdu 610051, China.

4. School of Medicine, Kunming University, Kunming 650214, China.

**Supplementary Methods**

**Bioinformatics analysis of SH3PXD2B expression in HCC tissues from the Human Protein Atlas database**

There are 6 hepatocellular carcinoma (HCC) cases and 3 non-tumor liver cases with their tissue sections stained with anti-SH3PXD2B available in the Human Protein Atlas database (<https://www.proteinatlas.org/>). These tissue sections were evaluated for their morphological characters by pathologists (X.K and Y.W) and downloaded. The levels of SH3PXD2B expression were analyzed using the Image-Pro Plus 6.0, Media, Cybernetics and expressed as the mean optical density (MOD). The raw data of individual cases were shown in Supplementary Table 1. The difference between the HCC and non-tumor liver tissue groups was analyzed by the Student’s t-test.

**The scoring system for immunohistochemical staining**

Individual tissue sections were scored by two trained pathologists (X.K and Y.W) in a blinded manner, based on the signal intensity as negative, weak positive, moderate positive, and strong positive.

Supplementary Table 1

The demographic information and mean optical density (MOD) of anti-SH3PXD2B staining in individual sections from the Human Protein Atlas database.

| Patient ID | Tissue | Gender | Age | MOD |
| --- | --- | --- | --- | --- |
| 2177 | HCC | Female | 58 | 18880 |
| 2280 | HCC | Male | 80 | 21050 |
| 2556 | HCC | Male | 72 | 13817 |
| 2766 | HCC | Female | 73 | 27039 |
| 3196 | HCC | Male | 65 | 14140 |
| 3346 | HCC | Female | 73 | 13844 |
| 2429 | Non-tumor livre | Male | 55 | 10860 |
| 3222 | Non-tumor liver | Female | 63 | 10014 |
| 3402 | Non-tumor liver | Female | 54 | 9838 |


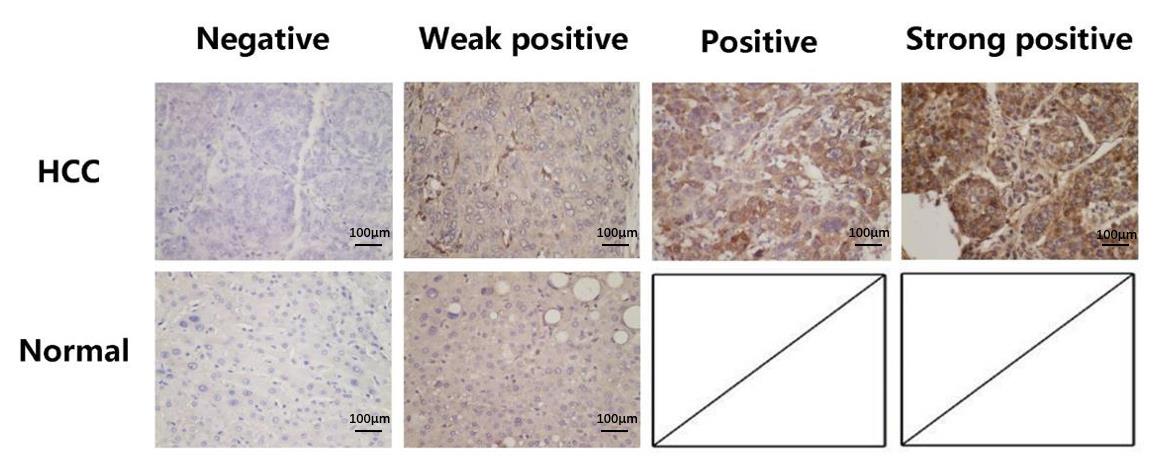


Supplementary Figure 1. Up-regulated SH3PXD2B protein expression in human HCC tissues. The levels of SH3PXD2B protein expression in 89 paired human HCC and non-tumor liver tissues were analyzed by IHC using anti-SH3PXD2B antibody. Data are representative images (magnification × 200) of each group from three separate experiments. Scale bar = 100 μm. The staining intensity was scored as four levels indicated.














Supplementary Figure 2. The original immunoblots using anti-SH3PXD2B in Figure 3.

(A and B) The representative immunoblots of Fig. 3A. (C and D) The representative immunoblots of Fig. 3C.
